# Dopamine Acts via D2-Like Receptors to Modulate Auditory Responses in the Inferior Colliculus

**DOI:** 10.1523/ENEURO.0350-19.2019

**Published:** 2019-10-11

**Authors:** Jeffrey M. Hoyt, David J. Perkel, Christine V. Portfors

**Affiliations:** 1 Integrative Physiology and Neuroscience and School of Biological Sciences, Washington State University Vancouver, Vancouver, WA 98686; 2 Departments of Biology and Otolaryngology, University of Washington, Seattle, WA 98195

**Keywords:** auditory, dopamine, inferior colliculus, mice, optogenetics, Parkinson’s

## Abstract

The ability to understand speech relies on accurate auditory processing of complex sounds. Individuals with Parkinson’s disease suffer from speech perception deficits, suggesting that dopamine is involved in the encoding of complex sounds. Recent studies have demonstrated that dopamine has heterogeneous effects on the responses of many neurons in the inferior colliculus (IC) of mice, although the strongest effect is to suppress neural activity. However, it was previously unknown which dopamine receptors are involved in modulating neuronal responses, and whether the observed preponderance of depressive effects reflects the endogenous dopamine system in the IC. In this study, we tested whether dopamine acts via D1- and/or D2-like receptors to alter responses of IC neurons in female and male mice. We also tested the effect of optogenetically induced dopamine release on auditory responses in the IC. We found that the effects of dopamine in the IC occur via D2-like receptors. In iontophoretic and freely behaving experiments, the single-unit and multi-unit effects of dopamine and a D2-like agonist were heterogeneous as both either increased or decreased responses of IC neurons to tones, while a D2-like antagonist had opposite effects. We also found that optogenetic activation of the endogenous dopamine system in the IC alters responses of auditory neurons. Similar to the effects of exogenous dopamine application, optogenetic induction of endogenous dopamine release heterogeneously altered auditory responses in the majority of cells in mice expressing channelrhodopsin-2 (ChR2). Understanding how dopamine modulates auditory processing will ultimately inform therapies targeting mechanisms underlying auditory-related communication disorders.

## Significance Statement

Understanding the neural mechanisms of normal and disrupted sound processing is a fundamental goal of auditory research. In this study, we examine the neuronal effects and underlying cellular and synaptic actions of dopamine on auditory processing in the auditory midbrain of normal-hearing mice. Our results increase our mechanistic understanding of auditory processing of sounds and how this encoding may change with different contexts, physiologic states, and disorders such as Parkinson’s disease. Understanding the mechanisms underlying normal dopaminergic modulation will help guide novel diagnostic and therapeutic approaches for disorders associated with abnormal dopamine signaling. Additionally, these studies using mice with normal hearing will facilitate future use of genetically modified mouse strains to target specific communication and neurologic disorders.

## Introduction

Communication through speech and language is a hallmark of human society and being unable to communicate can rapidly lead to social isolation and a high risk of depression (Medina and Weintraub, 2007; Monzani et al., 2008; Li et al., 2014; Dawes et al., 2015). Understanding language depends on appropriate extraction, differentiation, and representation of speech sounds by the auditory system (Belin et al., 2000; DeWitt and Rauschecker, 2012). However, communication disorders can arise from disruptions in the way individual neurons process sounds or in the ways neurons interact with each other. For example, when there is less of the neuromodulator dopamine in the brain, as occurs in individuals with Parkinson’s disease, neuronal responses to sound are altered and this results in speech processing disorders. Individuals with Parkinson’s disease have difficulties processing the timing (Gräber et al., 2002), loudness (Dromey and Adams, 2000; Ho et al., 2000), and emotional prosody (Dara et al., 2008; Schröder et al., 2010) of speech sounds, and even have problems determining speaker identity (Ariatti et al., 2008) and processing semantic information (Angwin et al., 2006). Combined, these studies support the notion of deficient speech processing in Parkinson’s patients, particularly in the dimensions of speech perception critical for everyday communication. Therefore, it is important to understand how dopamine normally regulates auditory processing to ultimately develop therapies to alleviate auditory disorders associated with dopamine.

Dopaminergic inputs and receptors reside in multiple auditory structures, including the inner ear, auditory brainstem, auditory thalamus, and auditory cortex (Bender et al., 2010; Happel et al., 2014; Toro et al., 2015; Nevue et al., 2016b). The primary auditory nucleus in the midbrain, the inferior colliculus (IC), also is rich in both dopaminergic fibers (Paloff and Usunoff, 2000; Tong et al., 2005) and D2-like dopamine receptors (Wamsley et al., 1989; Weiner et al., 1991). Moreover, dopamine injection into the IC affects auditory-evoked behavioral responses (Satake et al., 2012; Muthuraju et al., 2014; de Oliveira et al., 2014), indicating a functional role of dopamine in auditory processing in the IC. Recent studies have demonstrated that the sole source of dopaminergic input to the IC is the subparafascicular nucleus (SPF) of the thalamus (Nevue et al., 2016a), part of the A11 dopaminergic cell group (Takada et al., 1988). Although the SPF receives input from a variety of auditory structures, including the auditory cortex, auditory thalamus, the superior olivary complex, and the external and dorsal cortices of IC (LeDoux et al., 1985; Yasui et al., 1992; Wang et al., 2006), it is not known whether the SPF responds to sound. However, stimulation of SPF neurons evokes endogenous dopamine release in the IC (Batton et al., 2018). In addition, exogenous dopamine application has heterogeneous physiologic effects on IC neuronal responses to sound (Gittelman et al., 2013), but it remains unclear how dopamine produces these effects.

In this study, we tested whether (1) the heterogeneous effects of exogenously applied dopamine are also seen following activation of the endogenous dopamine system in the IC of mice, and (2) whether dopamine acts via D1-like and/or D2-like receptors to alter auditory responses of IC neurons. We compared the effects of exogenous dopamine application via iontophoresis with the effects of optogenetically induced endogenous dopamine release on response properties of single IC neurons. We also analyzed the effects of dopamine receptor subtype agonists and antagonists on single-unit and multi-unit responses in restrained and freely behaving mice. We found that both optogenetic stimulation of the endogenous dopamine system and exogenous dopamine application produce heterogeneous effects. We also found that D2-like receptors mediate the effects of dopamine on responses of IC neurons, and that the dopamine system actively modulates auditory processing in the IC. While the function of normal dopamine signaling in the IC is not well understood, our results provide a crucial first step toward understanding how dopamine exerts its effects on auditory processing, which may guide development of therapies for disordered dopamine signaling underlying communication-related and auditory-related neurologic disorders.

## Materials and Methods

### Subjects

We tested auditory responses in the central nucleus of the IC of 38 female and 37 male mice 1.5–7.5 months of age. Mice were group-housed in a vivarium with same-sex littermates on a reversed 12/12 h light/dark cycle schedule, and had *ad libitum* access to food and water. We performed all animal care and use procedures in accordance with the National Institutes of Health Guide for the Care and Use of Laboratory Animals, and the Institutional Animal Care and Use Committee of Washington State University, an AAALAC-accredited research institution. Beginning time of experiments in Zeitgeber time (ZT), where ZT0  =  lights on and ZT12  =  lights off, was approximately ZT17. Experimental sessions lasted no >5 h, and each mouse was used for one to four recording sessions. We monitored mice frequently, and if signs of distress were observed, the experiments were terminated. All founder breeder mice were purchased from The Jackson Laboratory. For iontophoretic and freely behaving experiments, we used CBA/CaJ mice (29 females and 29 males). For optogenetic experiments, we generated mice expressing the H134R variant of channelrhodopsin-2 (ChR2) selectively driven by the DA transporter (DAT) promoter of DA neurons by crossing B6.SJL-Slc6a3^tm1.1(cre)Bkmn^/J (DAT::Cre) heterozygous mice with B6;129S-Gt(ROSA)26Sor^tm32(CAG-COP4^*^H134R/EYFP)Hze^/J (Ai32(ChR2-YFP)) homozygous mice to create DAT:ChR2 mice (six females and four males) and wild-type littermates (three females and four males) on a C57Bl6/J background (O’Neill et al., 2017). Mice were genotyped by standard PCR procedures using a thermal cycler (G-Storm 482) according to the protocols published by Jackson.

### Surgical procedures

Preoperative analgesics included the NSAID meloxicam (2 mg/kg) administered subcutaneously immediately before surgery and the opioid buprenorphine (0.1 mg/kg) administered subcutaneously 1 h before surgery. We anesthetized mice with inhaled isoflurane (5% for induction and 1.5–2% for maintenance) and placed them in a stereotaxic apparatus. We administered an analgesic injection (1% lidocaine, 4 mg/kg) at the incision site, and attached a lightweight metal head post to the skull using dental cement (Muniak et al., 2012). A sharpened tungsten ground electrode then was cemented into the cerebellar or cerebral cortex on the opposite side of the IC hemisphere under investigation. To gain access to the brain, we made a craniotomy ∼2 × 2 mm in diameter aimed at the IC according to stereotaxic coordinates from an atlas of the mouse brain (Paxinos and Franklin, 2001). For iontophoretic and optogenetic experiments, we covered the craniotomy with bone wax. For freely behaving experiments, we additionally cemented a dual cannula (one plastic cannula to guide a recording electrode, and one metal cannula to guide a drug-delivery syringe) to the skull targeted over the IC, and lowered a tungsten recording electrode into and cemented it to the plastic cannula. We finally treated exposed tissue with topical application of lidocaine gel (3%) and triple antibiotic ointment (neomycin, polymyxin B, and bacitracin), and returned mice to the home environment for a recovery period of at least 24 h.

### Software accessibility

We controlled stimulus generation and data acquisition via custom-written software (Sparkle) run on an IBM PC computer with a Windows 7 Professional operating system. The code/software described in the paper is freely available online at https://github.com/portfors-lab/sparkle.

### Acoustic stimulation

We presented tone bursts (50-ms duration, 3-ms rise/fall time, 3/s) output through a 16-bit digital-to-analog converter (500,000 samples/s; National Instruments 2090A), sent through a power amplifier (Parasound HCA-1000A) to a programmable attenuator (Tucker Davis Technologies PA5), and routed to a free field speaker (LCY-K100). For iontophoretic and optogenetic experiments, we placed the speaker 10 cm from the ear contralateral to the IC under investigation. For freely behaving experiments, we mounted the speaker onto a plastic cage lined with sound attenuating foam. To confirm that stimuli did not contain artifacts, we calibrated the speaker output over a range of 5–110 kHz using a conditioning amplifier (Brüel & Kjær 2690) and calibrated microphone (Brüel & Kjær 4231) positioned at the location or height normally occupied by the mouse’s ear. Any stimulus distortion components were buried in the noise floor, at least 50 dB below the signal level, as determined by power spectral analysis of the microphone signals.

### Recording procedures

#### Iontophoretic experiments

We administered an intraperitoneal injection of acepromazine (2 mg/kg) to provide mild sedation, and then placed the mouse in a foam body mold and secured its head post to a stereotaxic device housed in a sound-attenuating chamber (Industrial Acoustics Company). We obtained extracellular recordings using glass pipettes (A-M Systems, 0.5- to 1-μm tip diameter, 20- to 30-MΩ resistance) filled with 1 M NaCl recording solution. For the application of pharmacologic agents, we constructed “piggyback” electrodes (Havey and Caspary, 1980) consisting of the recording pipette glued onto a multi-barreled glass pipette (20- to 25-μm total tip diameter). We controlled retention and ejection currents for each drug solution with a micro-iontophoresis unit (Dagan Industries). While searching for neurons, we applied a retention current of −15 nA to minimize drug leakage from the electrode tip. We advanced the electrodes in the brain using a hydraulic micropositioner (David Kopf Instruments 2650). Neuronal activity was amplified (Molecular Devices Multiclamp 700B), bandpass filtered (120–2000 Hz; Krohn-Hite 3364), and digitized by a 16-bit analog-to-digital converter (100,000 samples/s; National Instruments 2090A). We identified auditory-evoked responses by gradually advancing the electrode through the central nucleus of the IC while presenting pure tone and white noise bursts. After the isolation of a single unit and the recording of baseline responses to the stimulus set in the control condition, we ejected compounds with currents of +75 nA for a period of 5–20 min. We monitored raw waveforms on-line and stored them for off-line analysis.

**Figure 1.**
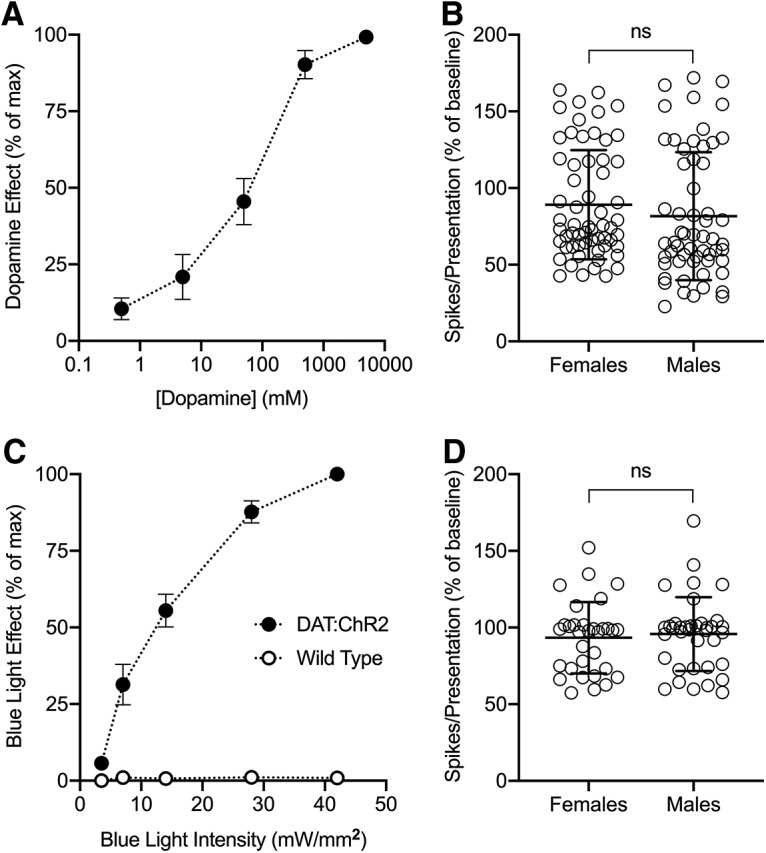
Postsynaptic and presynaptic manipulations of the dopamine system in the IC. ***A***, Exogenous dopamine had a dose-dependent effect on response rate, with robust effects occurring at 500 mM (*n* = 6). ***B***, Exogenous dopamine produced no magnitude or directional differences between sexes at 500 mM (*p* = 0.09*^a^*, *n* = 60 from females and 58 from males). ***C***, Endogenous dopamine had a dose-dependent effect on response rate, with robust effects occurring at 42 mW/mm^2^ in DAT:ChR2 mice (*n* = 15). Blue light had no effects in wild-type mice (*n* = 7). ***D***, Endogenous dopamine produced no magnitude or directional differences between sexes at 42 mW/mm^2^ (*p* = 0.59*^b^*, *n* = 32 from females and *n* = 37 from males). ns, not-significant.

To determine the optimal concentration of dopamine, we performed concentration curve experiments in which we constructed seven-barrel piggyback electrodes filled with five different concentrations of dopamine (0.5, 5, 50, 500, and 5000 mM). One of the remaining barrels was filled with 1 M NaCl and used as a sum channel to balance the currents. We randomly applied current through each barrel to *n* = 6 neurons, and allowed neuronal responses to recover to baseline between each concentration. We observed dose-dependent effects on response rate both in the increase (*n* = 2) and decrease (*n* = 4) directions. To evaluate the magnitude of the effects of different dopamine concentrations independent of directional effect, we calculated absolute percentage change relative to baseline at each concentration divided by the maximum percentage change in each neuron. We observed robust effects of dopamine on response rate at 500 mM (Fig. 1*A*). We did not observe significant magnitude or directional differences between sexes at this concentration (Fig. 1*B*, *p* = 0.09*^a^*).

We also used dopamine at this concentration (500 mM) as a reference point to evaluate the specificity of the dopamine receptor agonists and antagonists of interest. Since dopamine receptor agonists produce effects that are the same as or similar to dopamine, and since dopamine receptor antagonists oppose the effects of dopamine, alternative effects suggested that the pharmaceutical of interest produced non-specific effects. When this occurred we adjusted concentrations until such effects were no longer observed. Final concentrations used throughout the study were: dopamine (500 mM), SKF-38393 (SKF; 10 mM), quinpirole (QP; 10 mM), SCH-23390 (5 mM), and eticlopride (EP; 10 mM). For the remainder of iontophoretic experiments, we constructed three-barrel piggyback electrodes and filled two of the three barrels of with combinations of pharmacologic agents (all from Sigma-Aldrich). One barrel always contained dopamine, while the other barrel contained either the D1 agonist SKF hydrobromide, the D2 agonist QP hydrochloride, the D1 antagonist SCH-23390 hydrochloride, or the D2 antagonist EP hydrochloride. The remaining barrel was filled with 1 M NaCl and used as a sum channel to balance the currents.

#### Optogenetic experiments

To evoke the release of endogenous dopamine in the IC and record responses of single units before and after the dopamine release, we used either a custom-built optrode consisting of a microelectrode coupled to a glass fiber (Thomas Recording GmbH, 2.5-μm tip diameter, 10-MΩ resistance), or a glass fiber fed into the tip of a glass pipette (A-M Systems, 0.5- to 1-μm tip diameter, 20- to 30-MΩ resistance) via the Optopatcher (A-M Systems). Other than different electrodes, and stimulating the release of dopamine via blue light instead of applying exogenous dopamine iontophoretically, the optogenetic experiments used the same calibrations, recording equipment, and recording protocols as in the iontophoretic experiments.

To determine the optimal light stimulation parameters, we varied the pulse duration and wavelength of blue light (470 nm) through an optrode until we observed effects on response rate, with the most robust effects occurring with 75-ms pulses delivered at 6 Hz. We then performed intensity curve experiments in which we randomly stimulated at five different intensities of blue light (3.5, 7, 14, 28, and 42 mW/mm^2^) for each neuron, and allowed neuronal responses to recover to baseline between each intensity. To verify that effects were dependent on not only light intensity but also opsin expression, we recorded single units in both DAT:ChR2 (*n* = 15) and wild-type littermate (*n* = 7) mice. Methods to compute effect magnitude as a function of different light intensities were the same as those in iontophoretic experiments, with the most robust effects occurring in DAT:ChR2 mice at 42 mW/mm^2^; no effects were observed in wild-type mice (Fig. 1*C*). As was the case in iontophoretic experiments, we saw no significant magnitude or directional differences between sexes at this intensity (Fig. 1*D*, *p* = 0.59*^b^*).

At the end of the recording session, the mouse was deeply anesthetized with isoflurane in an induction chamber. We then transcardially perfused the animal using 60 ml of neutral buffered 10% formalin, removed the brain, and cryoprotected tissue overnight in 20% sucrose solution in 0.2 M sodium phosphate buffer. We sectioned the IC coronally at a thickness of 50 µm using a freezing microtome (Leica Biosystems SM2000R), and collected sections serially in 0.1 M phosphate buffer. We rinsed sections three times for 5 min in 0.1 M PBS, incubated sections in 50% ethanol for 30 min to permeabilize cell membranes, and again rinsed sections three times for 5 min in 0.1 M PBS. We then incubated sections for 1 h in blocking buffer [3% normal donkey serum (Millipore) in PBS], and rinsed sections three times for 10 min in 0.1 M PBS. We then incubated sections for 2 h in a solution of rabbit anti-GFP (1:500, Millipore) and 2% normal donkey serum in PBS. We mounted sections on Superfrost Plus microscope slides (Fisher Scientific), dehydrated and cleared sections, and coverslipped with DPX (Electron Microscopy Sciences). We observed labeling using a confocal microscope (Leica Microsystems TCS SP8).

#### Freely behaving experiments

We briefly anesthetized the experimental mouse in the induction chamber (∼5 min) using isoflurane gas (5%) to allow for attachment of lightweight wires to the recording electrode (A-M Systems, 0.5- to 1-μm tip diameter, 12-MΩ resistance) and ground pin implanted during surgical procedures. We then placed the mouse into a testing box (45 × 25 cm) lined with foam and fashioned with a flush-mount speaker, and placed the testing box inside a sound-attenuating booth (Industrial Acoustics Company). Recording and calibration equipment and parameters were the same as in iontophoretic and optogenetic experiments except that neuronal responses were acquired using a different preamplifier (Dagan Industries). The length and weight of the attached wires allowed the mouse to behave freely in the testing box. We monitored the behavior of the mouse with a computer camera (Logitech C615).

Recording began 10 min after the mouse had recovered from anesthesia and acclimated to the testing box. After recording baseline responses to different sounds from populations of neurons, we removed mice from the testing box and briefly anesthetized them (∼5 min) using isoflurane gas (5%) to allow for pressure injection of pharmacological agents (1 μl administered over the course of ∼1.5 min). Since we verified via iontophoretic controls that observed effects were due to pharmacological manipulations and not due to non-specific effects of the drugs or drug delivery, we used the same concentrations of dopamine (500 mM), QP (10 mM), and EP (10 mM) in freely behaving experiments. We then returned mice to the testing box and allowed them to recover. We monitored multi-unit responses over the course of ∼1 h following drug application. To verify that effects were due to receptor manipulation and not damage from drug delivery, we recorded from mice over multiple sessions using pressure injection of dopamine, agonists/antagonists, or vehicle.

### Experimental design and statistical analysis

To counterbalance between sexes, we non-randomly selected 38 female and 37 male mice for the study. We analyzed recorded waveforms and detected spiking events using a threshold feature that reliably separated single-unit or multi-unit activity with waveforms from the noise floor (Sparkle). We determined the characteristic frequency (CF; defined as the frequency requiring the lowest sound level to elicit an evoked response to 50% of the stimulus presentations) and threshold (MT; defined as the lowest intensity required to elicit a response to 50% of the stimulus presentations at CF) for each unit. We measured rate, probability (defined as percentage of presentations that elicited at least one action potential), mean first spike latency, and jitter (defined as the SD of first spike latency) in response to CF tone bursts at 10 dB above MT before and after application of pharmacological agents or stimulation with blue light.

Statistics were applied using either SSHF (Sparkle) or graphing software used to generate data plots (Prism, GraphPad). We performed the Shapiro–Wilk test to determine whether each data set fit a normal distribution, and evaluated whether all data sets in a group of analyses met this assumption. Because there were no instances in which data from all sets of analyses were normally distributed, for consistency we used either the Mann–Whitney *U* or the Wilcoxon matched-pairs signed rank test to assess statistical significance in all cases. Details for each statistical test are provided in Table 1. The critical significance level for each test was set at α = 0.05 (*p < 0.05, **p < 0.01, ***p < 0.001). For exemplar neurons and dose-response curves we computed within-subjects effects of dopamine manipulation, and values in the text represent the sample mean and SEM. For population analyses, we computed the fractional change caused by drug or light treatment in the response to sound. Values in the text represent the population mean and SD. Because we did not observe any sex differences during either iontophoretic or optogenetic experiments (Fig. 1*B*,*D*), we pooled data from both sexes for analyses.

**Table 1.** Statistical table

	Data structure	Type of test	Power
*a*	Not normal	Mann–Whitney *U*	95.05% CI –20.1 to 1.68
*b*	Not normal	Mann–Whitney *U*	95.13% CI –5.29 to 12.53
*c*	Not normal	Wilcoxon signed rank	96.48% CI –1.00 to –1.00
*d*	Not normal	Wilcoxon signed rank	96.48% CI 0.00 to 1.00
*e*	Normal	Wilcoxon signed rank	95.90% CI 9.09 to 20.21
*f*	Not normal	Wilcoxon signed rank	97.75% CI –13.64 to 0.00
*g*	Normal	Wilcoxon signed rank	95.90% CI –39.50 to –21.35
*h*	Not normal	Wilcoxon signed rank	95.90% CI –80.79 to –108.47
*i*	Not normal	Wilcoxon signed rank	95.90% CI –39.50 to –21.35
*j*	Normal	Wilcoxon signed rank	97.75% CI 10.34 to 45.57
*k*	Not normal	Wilcoxon signed rank	96.48% CI 0.00 to 0.00
*l*	Not normal	Wilcoxon signed rank	96.15% CI 3.02 to 3.98
*m*	Not normal	Wilcoxon signed rank	96.48% CI 0.00 to 0.00
*n*	Not normal	Wilcoxon signed rank	95.25% CI –2.58 to –1.06
*o*	Normal	Wilcoxon signed rank	97.73% CI 25.92 to 37.44
*p*	Normal	Wilcoxon signed rank	96.14% CI –40.80 to –18.87
*q*	Not normal	Wilcoxon signed rank	97.73% CI 1.00 to 18.56
*r*	Normal	Wilcoxon signed rank	96.14% CI –14.29 to 0.00
*s*	Not normal	Wilcoxon signed rank	97.73% CI –25.39 to –8.93
*t*	Normal	Wilcoxon signed rank	97.73% CI –57.86 to –13.13
*u*	Not normal	Wilcoxon signed rank	96.14% CI 6.88 to 26.76
*v*	Normal	Wilcoxon signed rank	96.14% CI 6.14 to 47.44
*w*	Not normal	Wilcoxon signed rank	95.72% CI –0.65 to 1.80
*x*	Not normal	Wilcoxon signed rank	95.72% CI 0.00 to 1.01
*y*	Normal	Wilcoxon signed rank	95.72% CI –1.96 to 0.85
*z*	Normal	Wilcoxon signed rank	95.72% CI –0.97 to 6.31
*aa*	Not normal	Wilcoxon signed rank	96.48% CI –1.00 to 0.00
*bb*	Not normal	Wilcoxon signed rank	96.48% CI 0.00 to 0.00
*cc*	Not normal	Wilcoxon signed rank	96.48% CI 0.00 to 0.00
*dd*	Not normal	Wilcoxon signed rank	96.48% CI –2.00 to –1.00
*ee*	Not normal	Wilcoxon signed rank	96.48% CI –51.14 to –28.02
*ff*	Not normal	Wilcoxon signed rank	96.48% CI –51.75 to –30.69
*gg*	Not normal	Wilcoxon signed rank	96.09% CI –4.89 to 1.73
*hh*	Not normal	Wilcoxon signed rank	96.09% CI –50.51 to –34.15
*ii*	Not normal	Wilcoxon signed rank	97.85% CI –57.02 to –38.42
*jj*	Not normal	Wilcoxon signed rank	97.85% CI –26.80 to –8.45
*kk*	Not normal	Wilcoxon signed rank	99.22% CI –59.51 to –20.93
*ll*	Not normal	Wilcoxon signed rank	99.22% CI –1.98 to 6.42
*mm*	Not normal	Wilcoxon signed rank	96.09% CI –39.56 to –20.18
*nn*	Not normal	Wilcoxon signed rank	96.09% CI –27.27 to –9.40
*oo*	Not normal	Wilcoxon signed rank	98.44% CI –2.66 to 3.87
*pp*	Not normal	Wilcoxon signed rank	98.44% CI –37.04 to –17.01
*qq*	Not normal	Wilcoxon signed rank	96.00% CI –4.00 to –3.00
*rr*	Not normal	Wilcoxon signed rank	96.00% CI –3.00 to 0.00
*ss*	Not normal	Wilcoxon signed rank	96.00% CI –1.00 to 1.00
*tt*	Not normal	Wilcoxon signed rank	96.00% CI 2.00 to 3.00
*uu*	Not normal	Wilcoxon signed rank	96.00% CI 3.00 to 4.00
*vv*	Not normal	Wilcoxon signed rank	96.00% CI –2.00 to 0.00

## Results

We recorded tone-evoked activity from 245 single-units and three multi-unit sites (∼5–10 units at each site) to investigate how dopamine affects auditory response properties in the IC. Recordings were conducted before, during, and when possible, following the application of either dopamine (*n* = 118; 60 from females and 58 from males), blue light pulses (*n* = 69; 32 from females and 37 from males), or dopamine plus selective D1 and D2 receptor agonists or antagonist (*n* = 58; 30 from females and 28 from males). We targeted units where the tone-evoked response could be clearly distinguished from spontaneous activity, and observed spiking patterns resembling those previously reported in the central nucleus of the IC including onset, sustained, pauser, and chopper-like responses (Wallace et al., 2012).

### Pharmacological application of dopamine has heterogeneous effects on response rate

**Figure 2.**
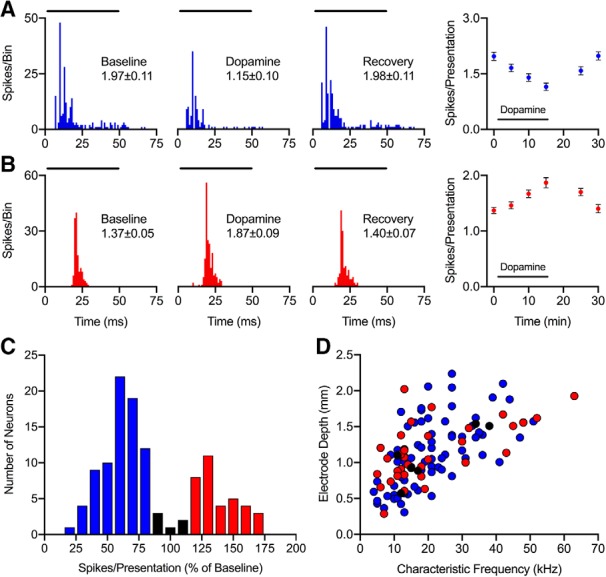
Exogenous dopamine application heterogeneously alters evoked spiking rate of IC neurons. ***A***, Representative example neuron in which iontophoretic dopamine application decreased the firing rate (*p* = 4.74 × 10^−7^*^c^*). The first three panels show peristimulus time histograms (PSTHs) of the response before (left), during (middle), and following (right) the delivery of dopamine. The horizontal bars above each PSTH denote the duration of the 27-kHz CF tone presented 100 times at 10 dB above threshold. The last panel represents the spiking rate at time points over the course of the recording protocol, with the time course of dopamine application depicted as a bar below the data points. ***B***, Representative example neuron that showed increased firing rate during iontophoretic dopamine application (*p* = 2.37 × 10^−5^*^d^*). Same conventions as in A except that the stimulus was a 43-kHz tone. ***C***, Normalized change in response rate during dopamine application (*n* = 118). Values >110% represent increases in spiking (red bars, *n* = 35), values <90% represent decreases (blue bars, *n* = 77), and values between 90% and 110% represent no effect of dopamine (black bars, *n* = 6). ***D***, Relationship between electrode depth and CF. Same neurons and color-coding conventions were used as in ***C***.

As has been shown previously (Gittelman et al., 2013), iontophoretic application of dopamine into the IC of awake mice produced mixed effects on evoked spiking rate. Neuronal responses could either decrease (Fig. 2*A*) or increase (Fig. 2*B*), but the majority of neurons showed decreased spike rate in the presence of exogenous dopamine (Fig. 2*C*). For each neuron, we calculated the mean spike rate during dopamine application normalized by the mean spike rate under control conditions and plotted the percentage effect of dopamine (Fig. 2*C*). For the remainder of the analyses, we maintained the color-coding relative to response rate, with blue signifying neurons in which dopamine caused a decrease and red signifying an increase in spiking. The majority of neurons (65%) showed a significant decrease in evoked firing following dopamine application (blue < 90%, *p* < 0.05), whereas 30% showed an increase in firing rate (red > 110%, *p* < 0.05). In the six neurons in which evoked firing changed by 10% or less, the changes were not significant (black = 90–110%, *p* > 0.05). We observed CF representation similar to previous reports in the IC (Saldaña and Merchán, 1992; Malmierca et al., 1995; Schreiner and Langner, 1997), with tonotopic organization of CFs ranging from 4 to 63 kHz. We did not observe a clear regional organization of increase versus decrease effects of dopamine in 118 neurons (Fig. 2*D*).

### Dopamine modulates the responsiveness and timing of IC neurons

**Figure 3.**
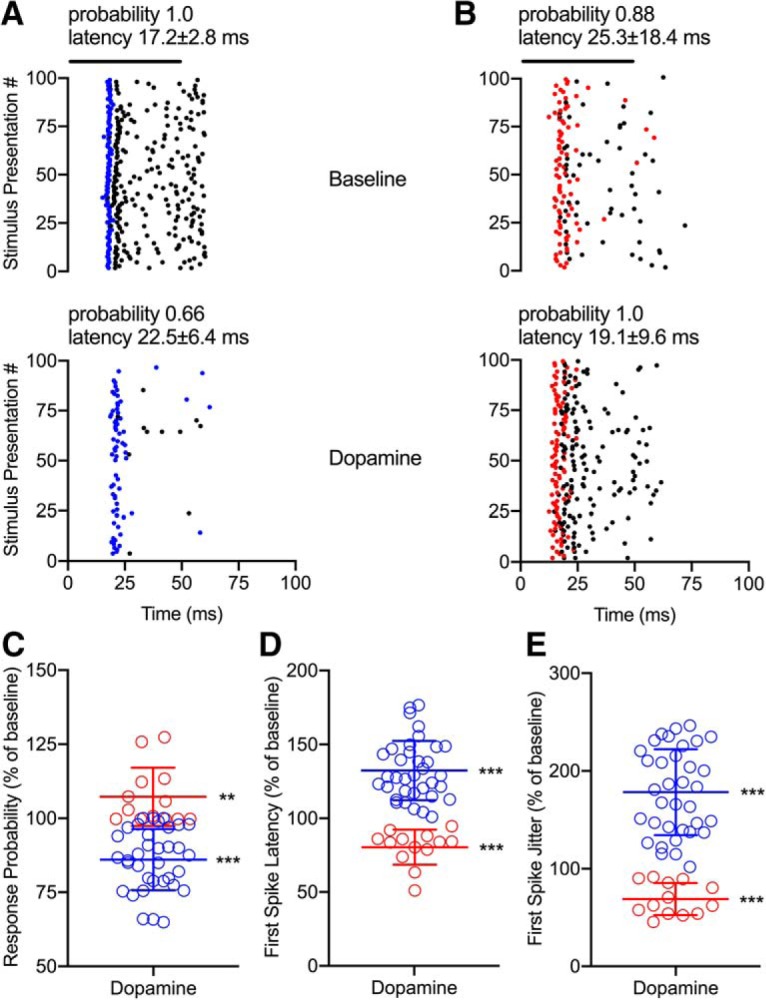
Effects of exogenous dopamine on response probability and timing reflect changes in spiking rate of IC neurons. ***A***, Raster plot of a representative example neuron where the 41-kHz CF tone was presented 100 times at 10 dB above threshold both before (top) and during dopamine application (bottom). Iontophoretic dopamine application decreased the number of CF presentations to which the neuron responded (*p* = 1.16 × 10^−10^*^k^*), and also increased the first spike (represented by blue dots) latency and jitter (*p* < 1 × 10^−15^*^l^*). Black dots represent subsequent spikes per presentation. ***B***, Same conventions as in ***A*** except the first spike per presentation is represented by red dots. Dopamine increased CF response probability (*p* = 4.88 × 10^−4^*^m^*) while decreasing both first spike latency and jitter (*p* = 1.67 × 10^−5^*^n^*). ***C****–****E***, Normalized change in response probability (***C***), first spike latency (***D***), and first spike jitter (***E***) during dopamine application (*n* = 48). Dopamine had the same directional effects on response rate and probability, while having opposite effects on response latency and jitter [compare red (*n* = 13) vs blue (*n* = 35)]. ** *p* < 0.005; *** *p* < 0.0005.

In a subset (*n* = 48) of the 118 neurons that changed their response rate with the application of dopamine, we obtained measures of response probability, latency, and jitter. Iontophoretic application of dopamine had heterogeneous effects as some neurons showed a decrease in probability and increase in response latency (Fig. 3*A*), while others displayed an increase in probability and decrease in latency (Fig. 3*B*). Overall, neurons that exhibited changes in evoked response rate during dopamine application also showed significant changes in response probability (Fig. 3*C*), where the direction of the effect on rate and probability were the same (compare blue circles vs red circles). Neurons in which dopamine caused a decrease in spiking rate also had a significant decrease in spiking probability (*p* = 4.66 × 10^−10^*^e^*), while neurons that increased in rate also increased in probability (*p* = 0.0078*^f^*). Additionally, neurons that displayed a decreased spiking rate during dopamine application (blue circles) showed a significant increase in first spike latency (Fig. 3*D*, *p* = 5.82 × 10^−11 ×^ *^g^*) and jitter (Fig. 3*E*, *p* = 5.82 × 10^−11^*^h^*), while neurons exhibiting an increased effect of dopamine on spike rate (red circles) had decreased response latency (*p* = 2.44 × 10^−4^*^i^*) and jitter (*p* = 2.44 × 10^−4^*^j^*).

### Optogenetic stimulation of the endogenous dopamine system produces effects similar to exogenous dopamine application

**Figure 4.**
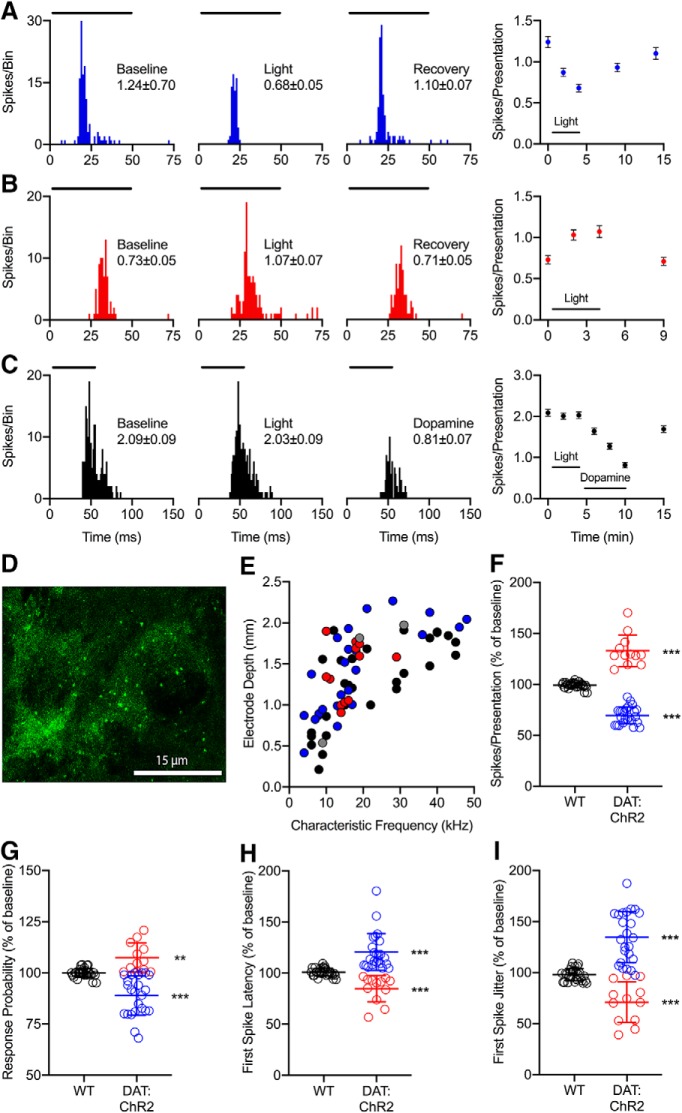
Effects of endogenous dopamine in the IC are similar to effects of exogenous dopamine application. ***A***, Representative example neuron from a DAT:ChR2 mouse in which optogenetically induced dopamine release decreased the firing rate (*p* = 4.83 × 10^−9^*^aa^*). Same conventions as in Figure 2 except that the stimulus was a 16-kHz tone and dopamine release was evoked with blue light pulses. ***B***, Representative example neuron from a DAT:ChR2 mouse that showed increased firing rate during stimulation with blue light (*p* = 1.71 × 10^−4^*^bb^*). Same conventions as in A except that the stimulus was a 10-kHz tone. ***C***, Representative example wild-type neuron that had no change in firing rate during stimulation with blue light (*p* = 0.45*^cc^*), although spiking was decreased by iontophoretic application of dopamine (*p* < 1 × 10^−15^*^dd^*). Similar conventions as in A and B except that the stimulus was a 17-kHz tone, and the rightmost peristimulus time histogram (PSTH) and subsequent quantification reflect the effect of dopamine. ***D***, Representative example coronal section of the IC from a DAT:ChR2 mouse, with DAT-Cre-EYFP-positive dopamine terminals labeled green. ***E***, Relationship between electrode depth and CF. In DAT:ChR2 mice, values >110% represent increases in spiking (red circles, *n* = 12), values <90% represent decreases (blue circles, *n* = 24), and values between 90% and 100% represent no effect of blue light (gray circles, *n* = 3). In wild-type mice, blue light stimulation produced no significant effect (black circles, *n* = 30). ***F***, Normalized change in response rate with blue light stimulation for the same neurons. In DAT:ChR2 mice, blue light pulses resulted in either a significant decrease (blue) or increase (red) in evoked response rate. In wild-type mice, blue light stimulation produced no significant effect (black). ***G****–****I***, Normalized change in response probability (***G***), first spike latency (***H***), and first spike jitter (***I***) during blue light stimulation for the same neurons. Similar to dopamine application, blue light had the same directional effects on response rate and probability, while having inverse effects on response latency and jitter in DAT:ChR2 mice (compare red vs blue). Pulsing blue light had no effect on these measures in wild-type mice (black). ** *p* < 0.005; *** *p* < 0.0005.

Consistent with results from iontophoretic experiments, optogenetically induced release of endogenous dopamine had heterogeneous effects on spiking rate (Fig. 4*A*,*B*). Importantly, application of blue light had no effect on neuronal responses in wild-type mice even when pharmacological activation of dopamine receptors did affect the neuronal response (Fig. 4*C*). We performed *post hoc* processing of IC sections from five DAT:ChR2 mice and four wild-type littermates to confirm the presence or absence of ChR2-EYFP. Dense labeling of ChR2-positive dopamine terminals was identified concentrated near IC cell bodies (Fig. 4*D*). We observed CF representation similar to iontophoretic experiments, with tonotopic organization of CFs ranging from 4 to 48 kHz. However, we did observe instances of regional organization of neurons exhibiting increase versus decrease effects of blue light stimulation; neurons that respond with an increase over a certain frequency range were found at a particular depth range (Fig. 4*E*). In DAT:ChR2 mice, 92% of neurons (*n* = 36/39) exhibited significant changes in evoked spike rate during blue light stimulation (Fig. 4*F*), with 62% showing a significant decrease (*p* = 1.19 × 10^−7^*^°^*) and 31% increasing in response rate (*p* = 4.88 × 10^−4^*^p^*).

In neurons where there was a significant effect of blue light on response rate to CF tones, we compared the effects of light stimulation on response probability, latency, and jitter. Consistent with results from exogenous application of dopamine, optogenetically induced dopamine release altered response probability in the same relative direction as response rate (Fig. 4*G*). Neurons that showed a decrease in rate (blue circles) additionally had a decrease in probability (*p* = 9.54 × 10^−7^*^q^*), while neurons that increased firing (red circles) also increased in response probability (*p* = 0.0039*^r^*). Also similar to iontophoretically applied dopamine, endogenous dopamine inversely altered response rate relative to response latency (Fig. 4*H*) and jitter (Fig. 4*I*). Neurons that displayed a decreased spiking rate during dopamine application showed a significant increase in first spike latency (*p* = 1.19 × 10^−7^*^s^*) and jitter (*p* = 3.58 × 10^−7^*^t^*), while neurons exhibiting an increased effect of dopamine on spike rate had decreased response latency (*p* = 4.88 × 10^−4^*^u^*) and jitter (*p* = 4.88 × 10^−4^*^v^*). We found no significant effects of blue light stimulation in *n* = 30 neurons from wild-type littermate mice (Fig. 4*F–I*; *p* = 0.21*^w^*, *p* = 0.83*^x^*, *p* = 0.35*^y^*, and *p* = 0.07*^z^*, respectively).

### D2-like receptors mediate the heterogeneous effects of dopamine in the IC

**Figure 5.**
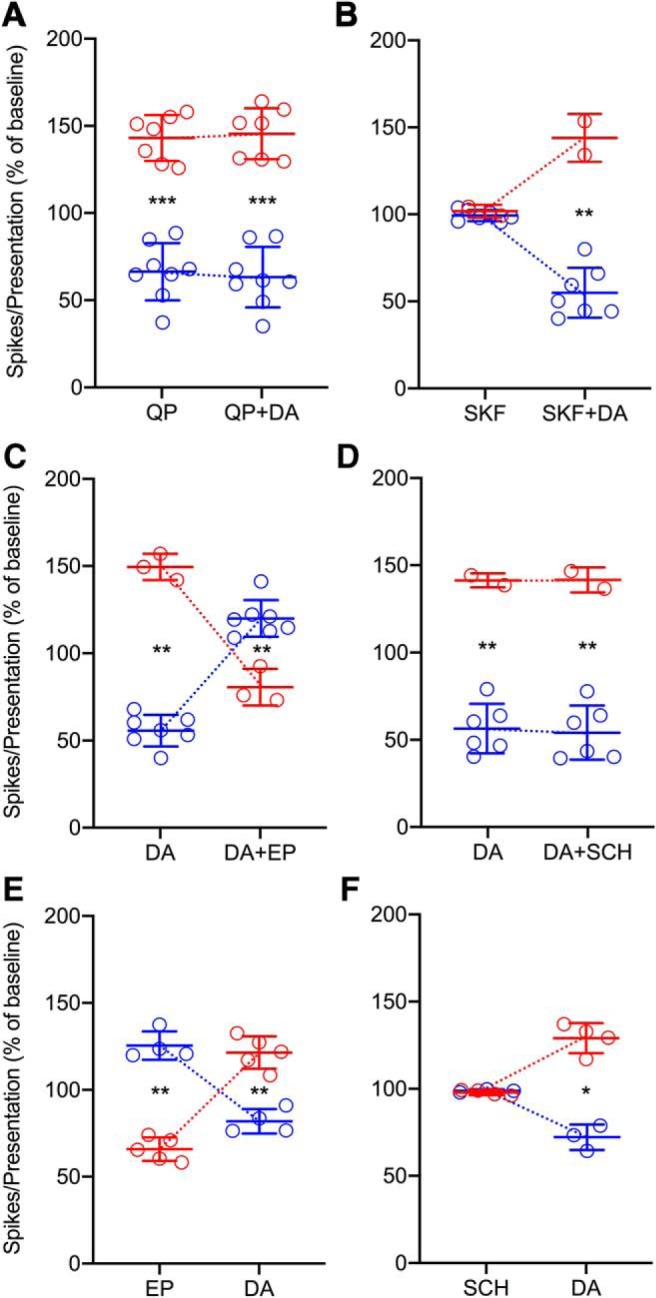
Heterogeneous effects of dopamine in the IC are mediated via D2-like dopamine receptors. ***A***, A D2 agonist (QP) affected spiking rate in the same direction and to a similar degree as dopamine (*n* = 15). ***B***, A D1 agonist (SKF) had no effect on response rate, but spiking was heterogeneously altered by dopamine (*n* = 9). ***C***, The heterogeneous effects of dopamine were directionally reversed beyond baseline spiking rate by a D2 antagonist (EP, *n* = 10). ***D***, The heterogeneous effects of dopamine on response rate were not affected by a D1 antagonist [SCH 39166 (SCH), *n* = 8]. ***E***, D2 antagonism with EP produced heterogeneous effects on evoked spiking, and dopamine reversed these effects beyond baseline rate (*n* = 9). ***F***, D1 antagonism with SCH had no effect on response rate, but dopamine did produce heterogeneous effects on spiking (*n* = 7). * *p* < 0.05; ** *p* < 0.005; *** *p* < 0.0005.

To determine the type(s) of dopamine receptors involved in the observed effects of exogenous and endogenous dopamine, we applied dopamine and selective D2/D1 agonists or antagonists to 58 of the original 245 neurons. Because of the length of the experimental paradigm applying multiple drugs, we focused our data collection and analyses only on response rate. The D2 agonist QP produced heterogeneous effects on auditory spiking rate (*p* = 6.10 × 10^−5^*^ee^*), and dopamine had effects in the same direction and to a similar degree (Fig. 5*A*, *p* = 6.10 × 10^−5^*^ff^*). The D1 agonist SKF had no effect on spiking rate (*p* = 0.18*^gg^*), but response rate was significantly altered by subsequent dopamine application (Fig. 5*B*, *p* = 0.0039*^hh^*). The heterogeneous effects of dopamine (*p* = 0.002*^ii^*) were reversed by the D2 antagonist EP, and this effect had a significant overshoot relative to baseline (Fig. 5*C*, *p* = 0.002*^jj^*). The effects of dopamine (*p* = 0.0078*^kk^*) were not affected by the D1 antagonist SCH (Fig. 5*D*, *p* = 0.11*^ll^*).

The observed overshoot given EP application suggested that the endogenous dopamine system might be contributing to baseline measurements. Indeed, initial application of EP produced heterogeneous effects on rate (*p* = 0.0039*^mm^*), and dopamine reversed these effects beyond baseline (Fig. 5*E*, *p* = 0.0039*^nn^*). Activity of endogenous dopamine might also result in maximum receptor occupancy of D1 receptors, which could account for the lack of effects given SKF application. However, SCH had no effect on response rate (*p* = 0.69*^oo^*), although spiking was altered by dopamine in these neurons (Fig. 5*F*, *p* = 0.016*^pp^*).

### Dopamine acts via D2-like receptors to modulate multiunit activity in the IC of freely behaving mice

**Figure 6.**
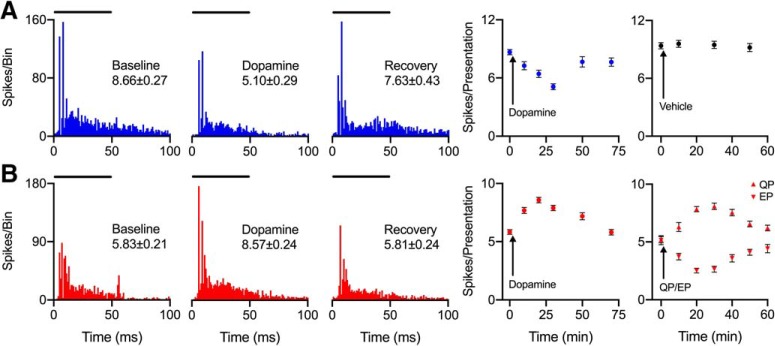
Effects of dopamine on multi-units in the IC of freely behaving mice reflect the effects of dopamine on single IC neurons. ***A***, Representative example multi-unit recording in which local dopamine injection decreased the firing rate. Same conventions as in Figures 2, 5 except that the stimulus was a 12-kHz CF tone presented 200 times. The last two panels represent the spiking rate at time points over the course of the recording protocol, with the delivery of dopamine (left) or vehicle (right) depicted as an arrow. ***B***, Representative example population of neurons that increased in firing rate given local dopamine injection. Same conventions as in A including that the stimulus coincidentally was also a 12-kHz tone, but the last panel represents spiking rate following injection of QP (upward triangles) or EP (downward triangles).

To confirm that results from iontophoretic and/or optogenetic experiments were not affected by restraint and/or sedative drugs, we injected dopamine into the IC of three freely behaving mice. Consistent with single-unit results from awake, yet sedated and restrained mice, dopamine altered multi-unit responses in the IC of normally behaving mice. The example in Figure 6*A* shows one of two populations of neurons that had a significant decrease in spiking rate following dopamine application (baseline, 8.66 ± 0.27 spikes/presentation; dopamine, 5.10 ± 0.29 spikes/presentation; *p* < 1 × 10^−15^*^qq^*). Although the global effect was a decreased response to dopamine injection, application of vehicle in a subsequent recording session produced no significant effects over multiple time points (baseline, 9.39 ± 0.32 spikes/presentation; minimum, 9.12 ± 0.42, *p* = 0.18*^rr^*; maximum, 9.58 ± 0.37, *p* = 0.84*^ss^*).

To confirm that results from experiments in single IC neurons reflect the activation/inhibition of D2-type dopamine receptors in the absence of acepromazine, a known allosteric inhibitor of D2 receptors (Kim et al., 2019), we compared three conditions (injection of dopamine, a D2 agonist, and a D2 antagonist) in the IC of a freely behaving mouse. Figure 6*B* shows that when dopamine increased multiunit response rate, the D2 agonist QP also increased response rate (baseline, 5.83 ± 0.21 spikes/presentation; dopamine, 8.57 ± 0.24; baseline, 5.19 ± 0.27; QP, 8.09 ± 0.17; *p* < 1 × 10^−15^ for both dopamine*^tt^* and QP*^uu^*). The D2 antagonist EP, however, had the opposite effect (baseline, 5.15 ± 0.39 spikes/presentation; EP, 2.53 ± 0.16 spike/presentation; *p* = 7.22 × 10^−9^*^vv^*). Clearly, these results indicate that the low level of acepromazine used in restrained experiments had no major effect on D2 receptors in the IC.

## Discussion

The IC receives dopaminergic projections from the SPF (Nevue et al., 2016a), contains D2-like receptors (Wamsley et al., 1989; Weiner et al., 1991), and application of exogenous dopamine to IC neurons can cause either increased or decreased neurophysiological responses (Gittelman et al., 2013). Our results here confirm these findings and expand on our understanding of how dopamine modulates auditory responses in the IC. Using optogenetics, we show that the release of endogenous dopamine also produces heterogeneous changes to neuronal responses in the IC. Using pharmacological approaches, we found that the heterogeneous changes in the IC are modulated by D2-like receptors and not D1 receptors. Application of dopamine in the IC could either increase or decrease sound-evoked firing rate, probability of firing, first spike latency, and first spike jitter. Application of a D2 agonist had similar effects on neuronal responses whereas application of either a D1 agonist or antagonist had no effect. In addition, application of a D2 antagonist altered response properties suggesting that the endogenous dopaminergic system was active during our recordings. Overall, our findings provide evidence that the dopamine system is an important modulator of auditory processing in the IC.

### Exogenous and endogenous dopamine produce similar heterogeneous effects on IC neurons

Pharmacologic and optogenetic manipulation of dopamine could increase or decrease sound-evoked firing rate, probability of firing, first spike latency, and first spike jitter of single neurons in the IC. Both endogenous and exogenous dopamine caused heterogeneous effects, where neuronal spiking rate and probability were relatively directionally similar and spiking latency and jitter were relatively opposite. Additionally, both types of dopamine manipulation revealed similar proportions of increased and decreased effects. Such similarities (or differences) were critical to establish, as iontophoretic application of pharmacological agents may lead to the spread of drugs to other neurons, potentially resulting in auditory and/or dopaminergic feedback loops (Saldaña and Merchán, 1992; Malmierca et al., 1995). Our results demonstrate that the effects of exogenous dopamine reflect the effects of endogenous dopamine in the IC.

### D2-like receptors mediate the heterogeneous effects of dopamine on IC neurons

Dopamine signaling relies on the interactions of dopamine with D1- and D2-like dopamine receptors, where D2-like receptors are typically depressive as they are coupled to the inhibitory G protein Gα_i/o_ (Spano et al., 1978; Sibley and Monsma, 1992). Consistent with previous studies showing that the IC expresses D2-like dopamine receptors (Wamsley et al., 1989; Weiner et al., 1991), our pharmacological results show that the effects of dopamine on neurons in the IC are mediated by D2-like receptors. Interestingly, pharmacological manipulation of D2-like receptors resulted in heterogeneous effects. Iontophoretic application of both dopamine and a D2 agonist increased or decreased auditory-evoked responses of single neurons to tone stimuli, while a D2 antagonist reversed the effects.

Further, the global effects of dopamine in the IC are in close agreement with the effects of dopamine on single neurons in the IC. Our results from multi-unit recordings in freely behaving mice show that injection of dopamine into the IC could increase or decrease the responses of populations of IC neurons. Activation of D2-like receptors using a D2 agonist produced effects similar to those of dopamine while application of a D2 antagonist had opposite effects. These results also provide evidence that lightly sedating the mouse with acepromazine, a known D2 antagonist (Monteiro et al., 2007), before single unit experiments and/or restraining the animal did not adversely affect the results.

In contrast to D2 receptors, D1-like dopamine receptors are typically facilitatory as they are coupled to the stimulatory G protein Gα_s/olf_ (Kebabian, 1978; Surmeier et al., 2007). While activation of D1-like receptors can induce a number of modulatory effects via different intracellular signaling cascades depending on cell type (Rangel-Barajas et al., 2015), we observed no effects of either a D1-like agonist or antagonist in 24 IC neurons. Interestingly, a recent study (Fyk-Kolodziej et al., 2015) found mRNA for each the D1-, D2-, D3-, and D5-specific dopamine receptor in the IC. This could be because gene expression does not necessarily result in protein expression. Another potential explanation is that D1-like receptors could be expressed on other types of IC neurons, for example visual (Mascetti and Strozzi, 1988) and/or somatosensory (Aitkin et al., 1981) neurons, and/or expressed in the external cortex of the IC and not in the central nucleus where we made our recordings.

Potential explanations for heterogeneous effects of dopamine on IC neurons following activation of a single type of dopamine receptor include activation of D2-like receptors inducing a number of different intracellular cascades. For example, activation of the D2 receptor can inhibit (Khurana et al., 2011) or stimulate (Vandecasteele et al., 2008) the hyperpolarization-activated (*I*_h_) cation current, which can increase or decrease the excitability of different cells, respectively. Additionally, D2 receptor activation can inhibit the calcium-activated potassium current (Ramanathan et al., 2008) or can stimulate the transient A-type potassium current (Perez et al., 2006), which can increase or decrease the excitability of different cells, respectively. Another possible explanation for our results is that activation of different types of D2-like dopamine receptors, for example D2-specfic versus D3-specific receptors, can produce opposing effects (Bryce and Floresco, 2019). Finally, the IC receives a number of excitatory and inhibitory auditory inputs (Saint Marie and Baker, 1990; Saint Marie, 1996; Merchán et al., 2005; Nakamoto et al., 2014; Buentello et al., 2015), and also contains an abundance of interneurons (Oliver et al., 1991). Thus, the presence of D2-like receptors on excitatory IC neurons and/or at excitatory synapses might result in the observed decreased effects, while activation of D2-like receptors on inhibitory IC neurons and/or at inhibitory synapses might produce a form of disinhibition, thereby producing the observed increased effects of dopamine. Understanding the precise mechanisms underlying each action of dopamine will require cellular and synaptic analyses.

### The endogenous dopamine system is active in the IC

Our data demonstrate a basal level of dopamine modulation occurring in IC neurons. In both restrained mice and freely behaving mice, we found that application of a D2-like antagonist following dopamine application resulted in an overshoot of spiking rate beyond that of baseline, and application of a D2-like antagonist produced heterogeneous effects that were opposite the effect of dopamine. These results are consistent with previous studies demonstrating that injection of a D2-like antagonist into the IC alters auditory-evoked potentials recorded in the IC (de Oliveira et al., 2014; Muthuraju et al., 2014). Additionally, iontophoretic application of a D2-like antagonist to IC neurons heterogeneously alters the probability of neuronal firing to novel auditory stimuli (Valdéz-Baizabal and Malmierca, 2018). Another recent study (Batton et al., 2018) used electrical and optogenetic stimulation of SPF neurons to evoke dopamine release in the IC, as measured by fast scan cyclic voltammetry. While the technique makes it possible to quantify dopamine release relative to baseline, it cannot measure baseline dopamine signaling. Investigations using inhibitory opsins would be helpful in quantifying basal dopamine levels in the IC.

These previous studies in conjunction with our results strongly suggest that basal activity of the endogenous dopamine system in the IC contributes to baseline measurements of IC neurons. However, given that optogenetic induction of endogenous dopamine release, as well as pharmacologic application of dopamine and/or D2-like agonists, can result in significant effects, it is evident that such basal dopamine activity does not produce maximal physiologic effects. Combined, these results indicate that the dopamine system can be further activated, and/or perhaps suppressed, likely given the activity of inputs to the SPF, the source of dopamine in the IC.

### Potential functional relevance of dopamine modulation in the IC

While behavioral studies are required to understand the precise functional role of dopamine signaling in the IC, understanding how dopamine modulates auditory responses of IC neurons is likely to be an integral part of understanding and interpreting results from behavioral studies. The IC contains both tyrosine hydroxylase-positive terminals (Tong et al., 2005) originating from the SPF (Nevue et al., 2016a), as well as D2-like dopamine receptors (Wamsley et al., 1989; Weiner et al., 1991), providing the machinery necessary for endogenous dopamine modulation in the IC. Based on work in other brain areas, endogenous dopamine levels can increase or decrease in response to novel or behaviorally salient stimuli, such as cues that predict reward or that occur in a social context (Aragona et al., 2003; Sasaki et al., 2006; Schultz 2010; Flagel et al., 2011). While these effects are associated with midbrain dopamine neurons, nothing is known about what makes SPF dopamine neurons fire. However, previous studies in the IC have shown that auditory responses are modulated by attention (Rinne et al., 2008) and reward-based learning (Metzger et al., 2006), suggesting that endogenous dopamine signaling in the IC may function to help modulate auditory processing depending on behavioral context; it would be advantageous for the auditory system to modulate its neuronal properties to selectively enhance processing of behaviorally relevant auditory stimuli while simultaneously reducing sensitivity to other auditory inputs, such as environmental sounds. Our finding that dopamine modulates neurophysiological response properties in the IC provides further evidence that endogenous dopamine may contribute to various forms of behavior-related modulation of sensory processing.

## Synthesis

Reviewing Editor: Darcy Kelley, Columbia University Decisions are customarily a result of the Reviewing Editor and the peer reviewers coming together and discussing their recommendations until a consensus is reached. When revisions are invited, a fact-based synthesis statement explaining their decision and outlining what is needed to prepare a revision will be listed below. The following reviewer(s) agreed to reveal their identity: NONE. Note: If this manuscript was transferred from JNeurosci and a decision was made to accept the manuscript without peer review, a brief statement to this effect will instead be what is listed below.

The manuscript has been responsive to the previous reviews and is a useful contribution to understanding dopamine modulation of auditory systems.

Some minor comments that should be addressed are:

Line 138: run not ran

Lines 275 - 277 What does approximately equal mean? Were the numbers of males and females similar but not exactly equal? Could just give the N's.

Lines 291 - 301 If data from all sets of analyses were not normally distributed, why use mean and SD as descriptors rather than, for example, medians and semi inter-quartile ranges?

Line 368 With regard to Fig. 4E, “regional organization” is vague. Neurons that respond with an increase over a certain frequency range were found at a particular depth range? Where are the 3 open grey circles that represent no effect of blue light?

Line 490 “converse to” is not grammatical. “In contrast to ..” or “Conversely”
